# A Comprehensive Physiologically Based Pharmacokinetic Framework of Ofloxacin: Predicting Disposition in Renal Impairment

**DOI:** 10.3390/pharmaceutics17091224

**Published:** 2025-09-20

**Authors:** Ammara Zamir, Muhammad Fawad Rasool, Iltaf Hussain, Sary Alsanea, Samiah A. Alhabardi, Faleh Alqahtani

**Affiliations:** 1 Department of Pharmacy Practice, Faculty of Pharmacy, Bahauddin Zakariya University, Multan 60800, Pakistan; ammarazamir20@gmail.com; 2 Center for Drug Safety and Policy, Xi’an Jiaotong University, Xi’an 710000, China; iltafhussain@stu.xjtu.edu.cn; 3 Department of Pharmacology and Toxicology, College of Pharmacy, King Saud University, Riyadh 11451, Saudi Arabia; salsanea@ksu.edu.sa; 4 Department of Pharmaceutics, College of Pharmacy, King Saud University, Riyadh 11451, Saudi Arabia; salhabardi@ksu.edu.sa

**Keywords:** PBPK, ofloxacin, renal impairment, AUC_0–t_, pharmacokinetics

## Abstract

**Background**: In the last several years, “physiologically based pharmacokinetic (PBPK) modeling” has gathered significant emphasis in the modeling of drug absorption, disposition, and metabolism. This research study aims to elaborate the plasma/serum concentration–time profiles and pharmacokinetics (PK) of ofloxacin by establishing a PBPK model in healthy subjects and those suffering from renal impairment (RI). **Methods**: A comprehensive literature analysis was conducted to screen out all the systemic PK profiles and parameters specific to ofloxacin, followed by their implementation in PK-Sim^®^ version 12 software. This model-driven approach begins by developing the model in healthy populations using both intravenous (IV) and per-oral (PO) routes and then extrapolating it to the diseased population. The model evaluation was then strengthened for different PK variables such as the maximal plasma/serum concentration (C_max_), the area under the curve from 0 to t (AUC_0–t_), and plasma/serum clearance (CL) by employing various metrics such as predicted/observed ratios (R_pre/obs_), visual predictive checks, the average fold error (AFE), root mean squared error (RMSE), and mean absolute error (MAE). **Results**: The AFE, RSME, and MAE for C_max_ in RI were 1.10, 0.22, and 0.16, respectively, which fell within the acceptable simulated error range. Furthermore, dosage adjustments for individuals with mild, moderate, and severe RI were presented by box-whisker plots to compare their systemic exposure with that of the healthy population. **Conclusions**: These model predictions have confirmed the PK variations in ofloxacin, which may assist the clinicians in optimizing dosage schedules in healthy and various categories of RI populations.

## 1. Introduction

Physiologically based pharmacokinetic (PBPK) modeling provides a precise estimation of the absorption, distribution, metabolism, and elimination (ADME) of drugs, thus playing a crucial role in their revelation and supporting decisions in the prediction of doses [1]. The recent research has highlighted the application of PBPK modeling in the prediction of the PK of various drugs in populations with renal impairment [2]. Additionally, the United States Food and Drug Administration (US FDA) has authorized the application of simulation techniques to reduce the need for in vivo studies, facilitating regulatory review, defining the risk assessment, and optimizing dosing in complex clinical conditions [3]. The combination of PBPK model-informed drug development and in vitro–in vivo extrapolation (IVIVE) has enabled researchers to depart from oversimplified interrupted approaches and comprehend the benefits of comprehensive modeling and dynamic simulation methods [4].

In contrast to compartmental modeling, the whole body is fractionated into various compartments depicting the real organs (liver, kidney, heart, stomach, spleen, etc.), tissues, and blood flows, which are interlinked via the circulating blood supply system in PBPK modeling [5]. The amalgam of physiological and compound-specific parameters, along with the PK information obtained via the plasma concentration vs. time curves, anticipates the disposition of drugs, making PBPK a decisive tool in individualized dosage regimens [3,6]. The advancements of these simulation tools focus their implementation on healthy, diseased (liver cirrhosis, chronic kidney disease, and congestive heart failure), and special populations (pediatrics, geriatrics, and pregnant women) [7]. Furthermore, PBPK modeling is used to forecast the PK of drugs in humans and manage serious drug–drug interactions by considering the impact of hepatic metabolism (recombinant enzymes, hepatic microsomes, and hepatocytes) [8]. A series of articles regarding PBPK modeling on different drugs has already been published in the last few decades [9,10,11,12,13].

Ofloxacin is a 4-quinolone antibacterial drug utilized for alleviating the symptoms of a wide variety of infections, such as respiratory tract, gynecological, skin and soft tissue, biliary tract, urinary tract, gonococcal, and non-gonococcal infections. [14]. Moreover, it is used off-label in the treatment of leprosy, traveler’s diarrhea, epididymitis, enteric fever, and spontaneous bacterial peritonitis [15]. Its mechanism of action involves the inhibition of bacterial topoisomerase II and IV, which interferes with three processes, i.e., DNA duplication, transcription, and repair, thus halting the bacterial cells’ division [15,16]. Ofloxacin is available in both intravenous (IV) and per-oral (PO) formulations at doses of 100 mg and 200 mg [17] as well as 300 mg and 400 mg [18,19,20], respectively. The Biopharmaceutics Classification System (BCS) has classified ofloxacin as class II, indicating its low solubility and high permeability [21]. Ofloxacin is reported to possess two acid dissociation constant (pKa) values, i.e., 6.05 (acidic) and 8.22 (basic) [22]. It is minimally metabolized in the liver, with 90% excreted unaltered in urine via two actions, i.e., glomerular filtration and active tubular secretion [23]. Ofloxacin has a black box warning of tendonitis and tendon rupture by the FDA [24] among elderly patients with renal impairment (RI).

Different pathophysiological changes, such as hematocrit, emptying time by the stomach, plasma protein scaling factor, and small intestinal transit time, occur in varying degrees of RI, as noted in the earlier peer-reviewed clinical publications [25,26]. These differences may influence the PK of ofloxacin, specifically its renal clearance (CL_R_), which decreases in RI; therefore, the integration of all these variations in the developed drug–disease PBPK model may give precise predictions for its disposition, thus resulting in optimal dosing among subjects with varying degrees of RI.

Currently, only a single conference abstract has been presented at the 2024 annual meeting, focusing on clinical ocular exposure among humans following the administration of ofloxacin ointment [27]. In contrast, another study has highlighted the quantification of the effect of different partition coefficient methods in PBPK modeling using various compounds, including ofloxacin [28]. Furthermore, many PBPK models on fluoroquinolone antibiotics have been developed, such as the impact on ciprofloxacin PK in pediatrics, geriatrics, and ICU patients [29,30] and determining the interindividual PK and pharmacodynamic variability of ciprofloxacin, levofloxacin, and moxifloxacin [31], etc. Still, no research article has discussed the development of the PBPK model in patients with RI. The novelty of our study is that no PBPK model of ofloxacin has been published to date, and this is the first one to address this gap in the literature by providing in-depth insights into ofloxacin PK among healthy subjects and the population with RI. This research investigation focuses on developing and analyzing a system-based PBPK model of ofloxacin in healthy (IV and PO routes) and diseased (RI) populations, which may be beneficial for health practitioners in improving dosage precision among these patients in clinical settings.

## 2. Materials and Methods

### 2.1. Screening of Reported Pharmacokinetic Data

A comprehensive search was conducted to extract clinical research studies on ofloxacin, comprising systemic plasma/serum concentrations versus time profiles after IV infusion and PO mode of administration in healthy subjects and those with RI via online search engines such as Google Scholar and PubMed. The studies were included based on the existence of data, i.e., age, weight, number of participants, percentage of females, dose, population, and administration route, as detailed in Table 1 below. Among the nine included profiles from six research publications, three individual profiles pertained to IV infusion, whereas eight were focused on per-oral administration. In addition, three profiles of RI (mild, moderate, and severe) were included in this study. These profiles were scanned to digitize them into numerical values by employing the GetData Graph Digitizer version 2.26 software. The PBPK model calibration was performed by utilizing one-third (1 IV infusion, 2 PO) of the studies, whereas two-thirds (2 IV infusion, 4 PO) were used in the verification, and all were used in the model’s final evaluation check. The parameter of the fraction unbound (f_u_) and specific intestinal permeability were optimized in the model calibration stage, whereas no parameters were calibrated further in the verification of the model.

### 2.2. Description of PBPK Simulation Software

The mechanistic PBPK framework for ofloxacin was developed by employing a whole-body PK simulation software, i.e., “PK-Sim^®^ Open System Pharmacology Suite (OSP)” version 12 (Bayer Technology Services, Biophysics, Germany) [34] to predict its ADME in normal subjects and those afflicted with RI (mild, moderate, and severe). The built-in physiological data related to age and anatomical changes in different populations in this database facilitates the refinement of incorporated parameters in the model.

### 2.3. Creation of Building Blocks

PK-Sim^®^ is a specialized software with a user-friendly graphical interface created by the OSP, which encompasses a variety of building blocks, including expression profiles, individuals, populations, formulations (in the case of PO route), administration protocols, events, and observed data. The relevant data on ofloxacin in various clinical conditions, i.e., healthy and diseased (RI), was collected from the reported literature to construct the building blocks. Furthermore, for the model structure, values of all physicochemical and drug-related parameters (solubility, fraction unbound, etc.) were compiled from the prior published scientific articles whose details are presented in Table 2.

### 2.4. Strategy for PBPK Model Building

**Figure 1:**
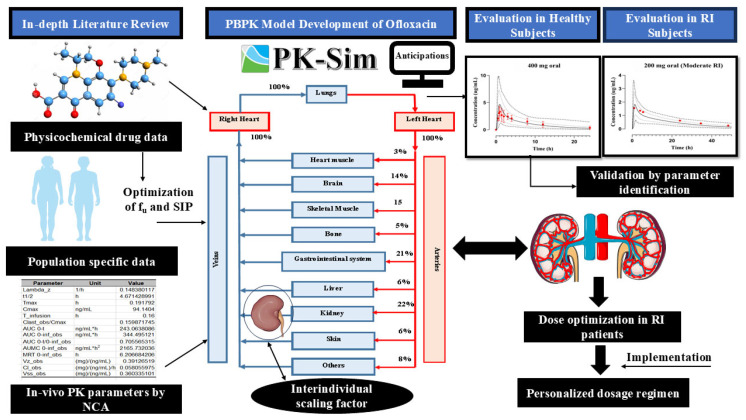
Schematic representation for constructing a PBPK model of ofloxacin. PBPK: physiologically based pharmacokinetic modeling; RI: renal impairment; PK: pharmacokinetics; NCA: non-compartment analysis; f_u_: fraction unbound; and SIP: specific intestinal permeability.

A methodical framework was employed for the creation of the mechanistic PBPK model, starting with an exhaustive search of scholarly publications to refine the systemic plasma/serum concentration–time profiles of ofloxacin. All the PK profiles, system-specific population data, and key input variables relevant to ofloxacin were then integrated into the “PK-Sim^®^ OSP suite” to develop the simulations in the healthy (IV infusion and PO route) population using previously established model-building procedures [9,40,41]. The IV model was created firstly to comprehend the baseline PK of ofloxacin to avoid the complicated processes of the PO route. After that, the PO model was subsequently developed by integrating the variable of specific intestinal permeability and the “dissolve” option from the formulation building block to forecast the absorption characteristics without modifying the other parameters used in the IV model. To further enhance the applicability of the developed PBPK model, it was then expanded to the population with differential grades of RI by utilizing the built-in population feature in the PK-Sim^®^ software. The graphical illustration of the modeling creation pathway is outlined in Figure 1.

### 2.5. Model Structure

Ofloxacin is designated with a chemical formula of C_18_H_20_FN_3_O_4_ [38] and pKa of 6.05 and 8.22 [22].

The lipophilicity (Log P) of 1.00 was utilized in the model, manually optimized from a range of values, i.e., −0.39 to 2.1 for ofloxacin. Furthermore, the value of f_u_ was optimized to 90% based on the predicted/observed ratios (R_pre/obs_) and visual predictive checks (VPC) from the two literature values of 80% [16] and 83% [39]. In PK-Sim software, models for absorption were built in, and the specific intestinal permeability was adjusted from a calculated value by PK-Sim, i.e., 4.92 × 10^−7^ cm/min to 4.92 × 10^−6^ cm/min due to the absence of any reported value in the literature. Sensitivity analysis was conducted for both of these parameters, and the results are presented in the Supplementary Figure S1 and Table S1. The “Rodger and Rowland” and “PK-Sim standard method” were employed to estimate the partition coefficient and cellular permeability. In addition, the value of ka (acidic phospholipids) under the heading of partition coefficient was scaled from 0.55 to 1.3 for simulations of RI. Moreover, the clearance (CL) of ofloxacin by glomerular filtration (CL_GF_) and tubular secretion (CL_TS_) was computed by formulas reported in the literature [42]. The formulas are as follows:

(1) $${CL}_{GF}=GFR\times f_{u}$$

(2) $${CL}_{TS}={CL}_{R}-{CL}_{GF}$$

The other determinants are specified in Table 2. Moreover, the details of drug and population-related input parameters are presented in the Supplementary file.

### 2.6. PBPK Model Structure in a Diseased Population

CKD is categorized into varied levels depending upon the severity of the RI by the “Kidney Disease Improving Global Outcomes” guidelines [25]. Based on eGFR, CKD is classified into four stages: mild, moderate, severe, and end-stage renal disease (ESRD). The eGFR of 40 mL/min/1.73 m^2^, 25 mL/min/1.73 m^2^, and 8 mL/min/1.73 m^2^ were embedded into the mild, moderate, and severe RI model as a mean from the presented range of values in the included study, while creating an individual in PK-Sim^®^ software [33], but the simulations were based on a population using a range of eGFR. Furthermore, a comparison of the area under the concentration vs. time curve from 0–t (AUC_0–t_) among the healthy, mild, moderate, and severe population after the PO route was undergone, followed by the visual depiction of box-whisker plots to suggest the precise doses of ofloxacin.

### 2.7. Evaluation of Model Predictions

A simulated cohort of approximately 1000 individuals was constructed using PK-Sim software to generate all systemic concentration–time profiles and demographic characteristics mentioned in the aforementioned studies. If data on demographics (age, weight, height, or body mass index) were missing, the configured default settings were integrated into the “PK-Sim^®^ OSP” database. The PBPK model of ofloxacin was then evaluated by VPC, where the anticipated dataset, along with the values of arithmetic mean, minimum and maximum concentrations and 5th to 95th centiles, was overlaid with the reported data. The Microsoft Excel Add-in program PK-Solver (version 2016) [43] was then utilized to perform the non-compartmental analysis (NCA) for the extraction of different PK variables such as CL, maximal plasma/serum concentration (C_max_), and AUC_0–t_ for expected and documented data. The “R_pre/obs_ along with 95% CI”, “mean R_pre/obs_”, “average fold error (AFE)”, “root mean squared error (RMSE)”, and “mean absolute error (MAE)” for all PK variables (C_max_, AUC_0–t_, and CL) were quantified to improve the evaluation of the model by using Equations (3)–(7), shown below. However, the R_pre/obs_ values in healthy IV were represented as a mean with a range due to only two accessible PK profiles.

(3) $$“R=\;\frac{Predicted\;value\;of\;PK\;parameter}{Observed\;value\;of\;PK\;parameter}”$$

(4) $$“\mathrm{Fold-error}=\;\frac{Predicted\;values\;of\;parameter}{Observed\;values\;of\;parameter}”$$

(5) $$“AFE=10^{\frac{\sum \log\left(fold\;error\right)}{N}}”$$

(6) $$“RMSE=\sqrt{{(1/n*\Sigma (\mathrm{simulated\_i}-\mathrm{observed\_i})\;}^{2}}”$$

(7) “MAE=1/n∗Σ|simulated_i−observed_i|”

## 3. Results

### 3.1. Assessment of the PBPK Model in a Healthy (IV and PO) Population

**Figure 2:**
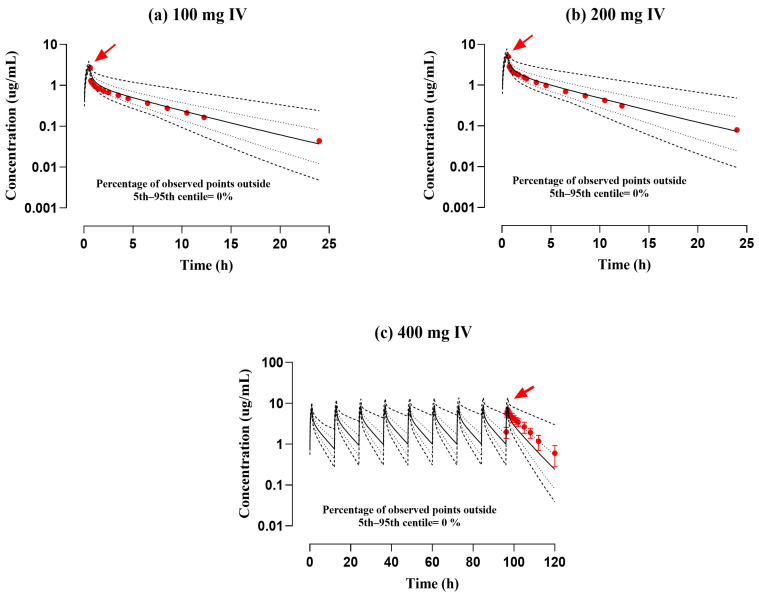
Documented and model-generated serum concentration profiles with respect to time after intravenous infusion administration in doses of (**a**) 100 mg [17], (**b**) 200 mg [17], and (**c**) 400 mg [32]. The published and simulated data records are illustrated by red round points and solid continuous lines, whereas minimum and maximum and 5th–95th centiles are visualized by dashed (---) and dotted (…) line formats, correspondingly. Observed data of profile (**c**) is portrayed along with the standard deviation after multiple dose administration, as mentioned in the published study. Red arrows indicate documented maximum plasma concentration (C_max_).

**Figure 3:**
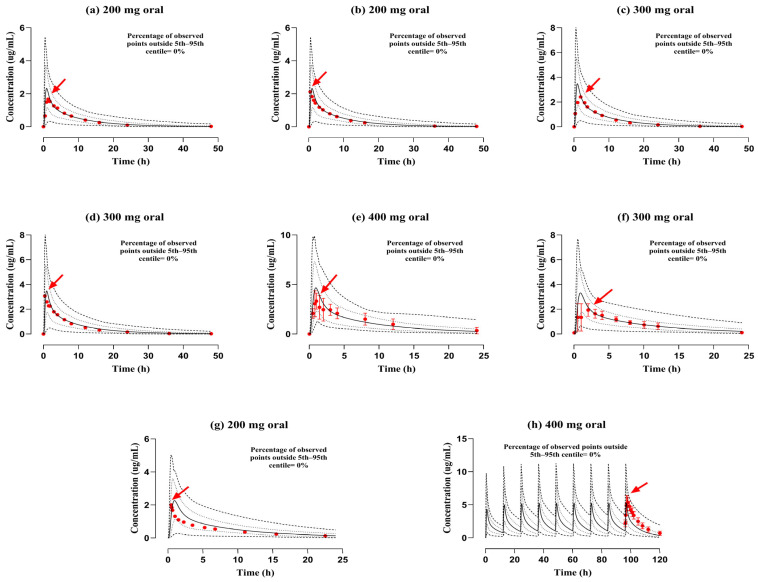
Documented and model-generated plasma concentration profiles with respect to time after per-oral administration in doses of (**a**,**b**) 200 mg [18], (**c**,**d**) 300 mg [18], (**e**) 400 mg [19], (**f**) 300 mg [20], (**g**) 200 mg [33], and (**h**) 400 mg [32]. The published and simulated data records are illustrated by red round points and solid continuous lines, whereas minimum and maximum and 5th–95th centiles are visualized by dashed (---) and dotted (…) line formats, correspondingly. Observed data of profiles (**e**), (**f**,**h**) are portrayed along with the standard deviation as mentioned in the published study. Red arrows indicate documented maximum plasma concentration (C_max_).

**Figure 4:**
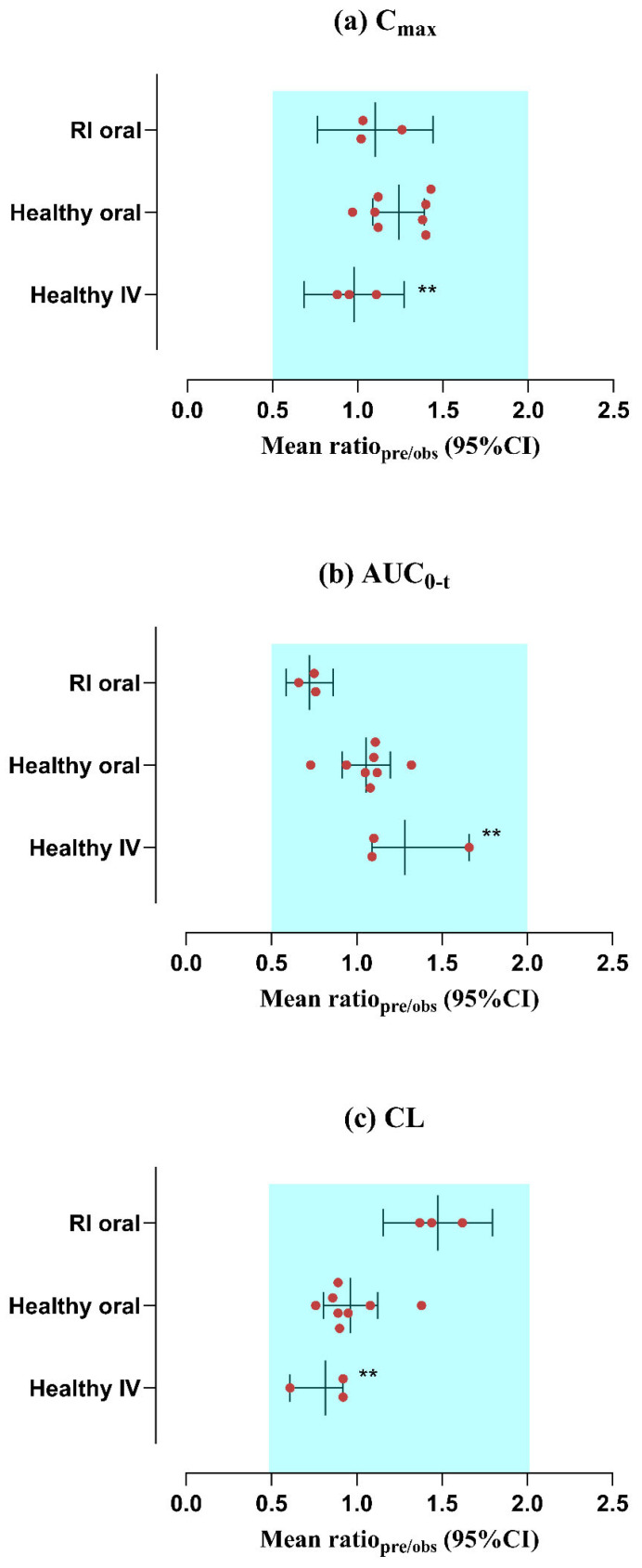
Mean (R_pre/obs_) comparison for PK endpoints such as (**a**) C_max_, (**b**) AUC_0–t_, (**c**) CL among healthy subjects (IV infusion and PO) and those with RI (PO)The study results are provided with a 95% confidence interval (CI). C_max_: maximal concentration of plasma/serum; IV: intravenous route of administration; AUC_0–t_: area under the curve from time 0 to t; CL: clearance; and RI: renal impairment. ** In healthy subjects following the IV infusion route, the findings are depicted as the mean with the range due to only three accessible profiles.

The reported and simulated data from plasma/serum concentration over time curves following IV infusion and PO application of ofloxacin in administered dosages ranging from 100–200 mg to 200–400 mg can be depicted in Figure 2 and Figure 3. The reported findings were closely identical to those of simulated graphs when compared with the 5th to 95th centiles. The “mean R_pre/obs_” ratios for C_max_, AUC_0–t_, and CL were computed and graphically presented in Figure 4. Moreover, the precision of the developed model of ofloxacin was further verified with AFE for all PK parameters. The AFE values for AUC_0–t_ were 1.28 (after the IV route) and 1.05 (after the PO route), indicating that they fall within the criteria of the 0.5–2 predictive fold error range. Additional metrics, such as RMSE and MAE, were calculated to enhance the evaluation. The RMSE and MAE values for C_max_ were 1.00 after the IV route, indicating that the model accurately represented the ADME of ofloxacin. The residual PK variables metrics are presented in Table 3 and Table 4

### 3.2. Evaluation of the PBPK Model in the Population with RI

**Figure 5:**
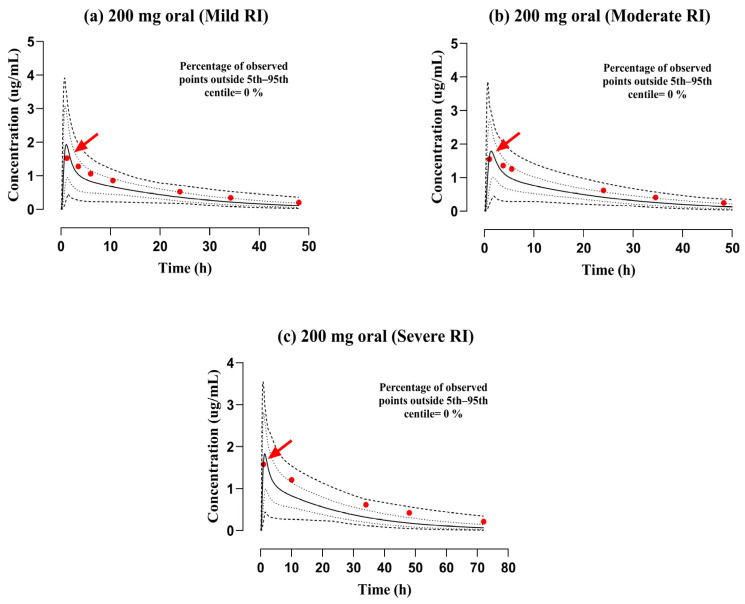
Documented and model-generated plasma concentration profiles with respect to time in subjects with mild, moderate, and severe RI (**a**–**c**) 200 mg [33]. The published and simulated data records are illustrated by red round points and solid continuous lines, whereas minimum and maximum and 5th–95th centiles are visualized by dashed (---) and dotted (…) line formats, correspondingly. Red arrows indicate documented maximum plasma concentration (C_max_). RI: renal impairment.

The mechanistic PBPK model for ofloxacin was developed among individuals with varying degrees of RI (mild, moderate, and severe), as outlined in the relevant study [33]. The observed data points were in concordance with the simulated ones after administering a 200 mg PO dose. The VPC for both data (reported and predicted), along with the 5th–95th centiles, is displayed in Figure 5. The AFE values were falling within a 2-fold error range based on the C_max_ of 1.10. Moreover, the values of RMSE and MAE for CL were 2.43 and 2.41, respectively, indicating that they are within the acceptable range (Table 4). The values of R_pre/obs_ for C_max_, AUC_0–t_, and CL are depicted in Table 5, and their graphical presentation is in Figure 4.

### 3.3. Dose Amendments in Subjects with RI

**Figure 6:**
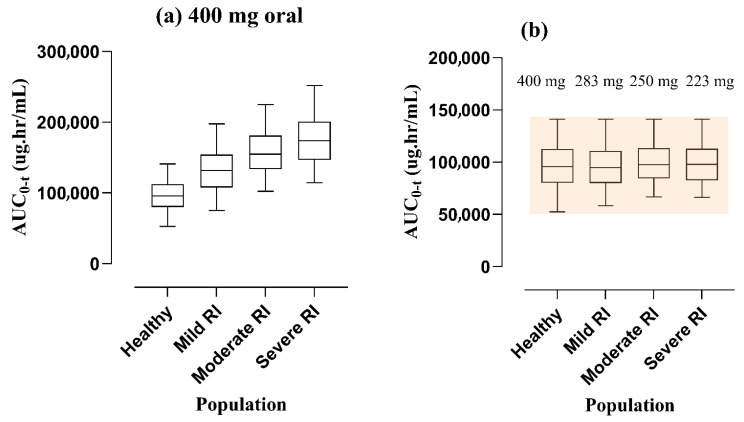
Visual depiction of predicted AUC_0–t_ alongside 5th–95th centile by utilizing box-whisker graphs after oral intake of a 200 mg dose (**a**) in the normal population and those with RI. The dose modification in mild, moderate, and severe populations is presented in (**b**). AUC_0–t_: area under the plasma concentration vs. time curve from time 0 to t; RI: renal impairment.

After creating and assessing the PBPK simulations of ofloxacin in healthy subjects and those suffering from RI, the AUC_0–t_ values among mild, moderate, and severe RI were depicted to be increased after the administration of similar doses via the PO route of administration. Consequently, to achieve the target of equal exposure for ofloxacin among both populations (healthy and RI), a step-by-step process was adapted to taper off the doses by conducting simulations with various administration protocols. The dose was reduced by 29.25%, 37.5%, and 44.25% in mild, moderate, and severe RI after the PO route, which is represented by box-whisker plots (See Figure 6). The entire process of dose amendment was adapted as per previously reported clinical research papers [44].

## 4. Discussion

This study has established the PBPK model for ofloxacin in healthy and RI populations after IV and PO dosage formulations for the first time by utilizing a methodical approach. The resultant PBPK model aims to forecast personalized dosage regimens for individuals with different levels of long-term disease (RI). Initially, the model for ofloxacin was developed using healthy participants, based on previously reported PBPK model-based research articles [9,10,11,12,13] to determine the underlying variations in every PK variable. The model was subsequently expanded to the diseased state, i.e., RI, by incorporating different pathophysiological alterations.

To explain the disposition of ofloxacin, a PBPK model was constructed by PK-Sim software, and the AUC_0–t_ values of the mean predicted data were slightly greater than that of observed data, i.e., 11.87 μg·h/mL vs. 10.83 μg·h/mL, after IV infusion administration of ofloxacin in healthy participants. Moreover, the mean simulated values after the PO route for C_max_ were 3.09 ug/mL, which was analogous to that of reported metrics, i.e., 2.41 μg/mL. The estimated AFE, RMSE, and MAE for ofloxacin CL following the PO route were 0.96, 1.89, and 1.6, respectively, indicating that the model has effectively captured the disposition of ofloxacin. Moreover, the predicted IV AUC_0–t_ at a dose of 200 mg, i.e., 15.81 μg·h/mL, is slightly less in comparison to that of PO AUC_0–t_, i.e., 16.07 μg·h/mL in the study by Stein et al. [18], which should be ideally more, as presented in another study by Fillastre et al. [33], i.e., 13.77 μg·h/mL vs. 15.81 μg·h/mL. These minor variations in AUC_0–t_ in the former study may result from interindividual differences. Moreover, a multiple-dose study by Flor et al. [32] has presented the predicted IV AUC_0–t_ of 270.57 μg·h/mL at a dose of 400 mg in comparison to the PO AUC_0–t_, i.e., 131.66 μg·h/mL.

In humans, ofloxacin is eliminated unaltered in urine, which accounts for ~90% of the administered dose, and partially through metabolism by the liver [23,45]. According to a reported study, [46] any drug that has >30% elimination from the body requires dosage modification in individuals with RI of a different severity. Moreover, the FDA label for ofloxacin has also mentioned dosage adjustments in subjects with RI who have a creatinine clearance (CL_CR_) less than or equal to 50 mL/min, and the dose should be halved if the CL_CR_ is less than 20 mL/min [47]. As ofloxacin is excreted mainly from the body in urine, its ADME may be significantly affected in patients with RI. Many changes in pathophysiology occur in the case of CKD, as recorded in previous studies [25,26] such as hematocrit, eGFR, etc.

The included study in the PBPK model consisted of three individual profiles belonging to the mild (CL_CR_ of 45.0 ± 4.8 mL/min/1.73 m^2^), moderate (CL_CR_ of 26.2 ± 4.4 mL/min/1.73 m^2^), and severe (CL_CR_ of 11.5 ± 3.0 mL/min/1.73 m^2^) RI, whose profiles are presented in the graphical depiction (Figure 4 and Figure 5). The simulated value of AUC_0–t_ was lower than the reported value, i.e., 33.56 μg·h/mL vs. 50.12 μg·h/mL. These findings suggest that alterations in RI increase the CL of ofloxacin. As the FDA has issued a black box warning regarding tendonitis in the elderly with RI, dosing decisions should be made by the physician, considering all aspects of the patient and carefully balancing the therapeutic margin. Furthermore, the systemic exposure of ofloxacin and its dosage schedule was anticipated in this drug–disease PBPK model after the administration of PO dosage forms. The AUC_0–t_ was decreased by about 29.25%, 37.5%, and 44.25% in mild, moderate, and severe RI via the PO route. The visual illustrations for the dose modifications of ofloxacin, employing box-whisker plots (See Figure 6), may help physicians circumvent the severity of the disease (RI).

The notable merit of this research study is that a PBPK model for ofloxacin has been developed for the first time, which has successfully modeled its ADME in healthy and diseased subjects (RI). There are some limitations of the study that should be considered. The plasma/serum concentration over time curves were scanned point by point to collect data for model evaluation. However, this method may not be as precise as the updated data, which could impact the accuracy of the results. The value of f_u_ was optimized based on mean R_pre/obs_ and VPC to improve the model’s applicability. Additionally, the specific intestinal permeability of ofloxacin was not available in the literature, so the value computed by PK-Sim software was further adjusted to establish the model. A challenge in this study was that the pattern of ofloxacin profiles in healthy and RI individuals across different studies was highly variable due to interindividual differences, preventing a consistent parameter setting. The access to more clinical studies on ofloxacin that account for this variability could facilitate the calibration of a PBPK model with better predictability.

## 5. Conclusions

The presented PBPK model has effectively predicted the PK of ofloxacin in healthy subjects and those with RI. Distinct disease-related variations were accounted for in the mild, moderate, and severe RI to augment the accuracy of the model. Considerable changes are depicted in the AUC_0–t_ among subjects with RI, suggesting the necessity for tailoring the doses of ofloxacin to mitigate the likelihood of undesirable drug reactions and, in turn, increasing the patient-centered efficacy of personalized treatment schedules.

## Figures and Tables

**Table 1:** Demographic characteristics and dosage schedules of included studies in the conception of the mechanistic PBPK model of ofloxacin.

Sr. No.	Study Reference	Target Population	No. of Enrolled Participants	Applied Dosage (mg)	Mode of Administration	Female Proportion (%)	Age (Years)	Weight (kg)
1-	Lode et al. (1988) [17] ^a,b^	Healthy	10	100	IV infusion	50	25–46	54–74
				200				
2-	Stein et al. (1991) [18] ^b^	Healthy	32	200	PO	0	18–37	59–99
				300				
3-	Sanchez Sánchez et al. (1994) [19] ^a^	Healthy	10	400	PO	20	21–50	62.3 ± 13.2
4-	Molinaro et al. (1992) [20] ^a^	Healthy	12	300	PO	0	20–27	62–86
5-	Flor et al. (1993) [32] ^b^	Healthy	20	400	IV infusion	0	18–41	61–100
					PO			
6-	Fillastre et al. (1987) [33] ^b^	Healthy	12	200	PO	N/M	27.7 ± 3.1	71 ± 6.2
		Mild RI					58.0 ± 11.9	63.6 ± 11.5
		Moderate RI					63.3 ± 8.2	72.4 ± 12.9
		Severe RI					63.6 ± 13.3	74.4 ± 20.5

IV: intravenous; RI: renal impairment; PO: per-oral; and N/M: not mentioned. ^a^ These studies were used in the model calibration. ^b^ These studies were used in the model verification.

**Table 2:** List of all input baseline parameters incorporated in the PBPK model calibration of ofloxacin.

Model Parameters	Incorporated Values	Reference ID
Physicochemical characteristics		
Molecular mass (g/mol)	361.388	[23]
pKa	pKa1 6.05	[22]
	pKa2 8.22	
Plasma protein binding	Human serum albumin	[35]
Solubility in water (mg/mL)	2.66 mg/mL @ pH 7	[36]
Log P (log units)	Range (−0.39–2.1) ^a^	[36,37,38]
Absorption		
Specific intestinal permeability (cm/min)	4.92 × 10^−6 b^	Optimized
Distribution		
Cellular permeability model	PK Sim Standard	[34]
Partition coefficient model	Rodger and Rowland	[34]
Specific organ permeability (cm/min)	1.29 × 10^−4^	Calculated by PK-Sim
Fraction of unbound drug (f_u_)	80%, 83% ^c^	[16,39]
Metabolism and elimination		
Total hepatic clearance (L/h/kg)	0.04	[33]
GFR fraction	0.90 ^d^	[33]
Tubular secretion (L/h)	5.52 ^e^	[33]

GFR: glomerular filtration rate; Log P: lipophilicity. ^a^ The Log P measurement of 1.00 is utilized within the range of values by manual optimization. ^b^ Value is optimized from the PK-Sim calculated value, confirmed by visual predictive checks. ^c^ Value is optimized to 90% based on visual predictive checks and R_pre/obs_. ^d^ Value is calculated from reported GFR and the clearance by the glomerular filtration (CL_GF_) process. ^e^ Value is calculated from renal clearance (CL_R_) and CL_GF_.

**Table 3:** R_pre/obs_ ratios for different PK variables of ofloxacin in healthy and RI populations.

Sr. No.	Study ID	Administered Doses	C_max_ (μg/mL)			AUC_0–t_ (μg·h/mL)			CL (L/h)		
			PRE	OBS	R Ratio	PRE	OBS	R Ratio	PRE	OBS	R Ratio
		Healthy population (IV infusion route)									
1-	Lode et al. (1988) [17]	100	2.39	2.7	0.88	7.94	7.19	1.1	12.16	13.19	0.92
2-	Lode et al. (1988) [17]	200	4.78	4.98	0.95	15.81	14.47	1.09	12.21	13.23	0.92
3-	Flor et al. (1993) [32]	400	7.74	6.92	1.11	253.68	152.05	1.66	1.56	2.52	0.61
		Healthy population (PO route)									
1-	Stein et al. (1991) [18]	200	2.33	1.66	1.4	16.07	14.39	1.11	12.3	13.71	0.89
2-	Stein et al. (1991) [18]	200	2.33	2.11	1.1	16.08	15.24	1.05	12.29	12.87	0.95
3-	Stein et al. (1991) [18]	300	3.46	2.41	1.43	22.7	20.2	1.12	13.2	14.67	0.89
4-	Stein et al. (1991) [18]	300	3.46	3.08	1.12	23.06	20.79	1.1	12.83	14.24	0.9
5-	Sanchez et al. (1994) [19]	400	4.64	3.31	1.4	27.9	29.49	0.94	12.99	12	1.08
6-	Molinaro et al. (1992) [20]	300	3.19	2.3	1.38	20.57	18.99	1.08	12.99	15.07	0.86
7-	Fillastre et al. (1987) [33]	200	2.25	2	1.12	13.77	10.42	1.32	12.95	16.98	0.76
8-	Flor et al. (1993) [32]	400	5.25	5.36	0.97	118.3	160.16	0.73	3.26	2.36	1.38

C_max_: maximal plasma concentration; IV: intravenous; AUC_0–t_: area under the plasma/serum concentration vs. time curve from 0–t; PRE: predicted; CL: clearance; OBS: observed; and PO: per-oral.

**Table 4:** Computation of average fold error values for PK variables in healthy and RI populations.

Population/Route	PK Variable	AFE	RMSE	MAE
Healthy adults (IV)	C_max_	0.98	0.51	0.44
	AUC_0–t_	1.28	58.68	34.57
	CL	0.81	1	1
Healthy adults (Oral)	C_max_	1.24	0.73	0.61
	AUC_0–t_	1.05	14.93	6.95
	CL	0.96	1.89	1.6
RI (Oral)	C_max_	1.1	0.22	0.16
	AUC_0–t_	0.71	12.47	11.31
	CL	1.54	2.43	2.41

C_max_: maximal plasma concentration; IV: intravenous; AUC_0–t_: area under the plasma/serum concentration vs. time curve from 0–t; CL: clearance; RI: renal impairment; PO: per-oral; AFE: average fold error; RMSE: root mean squared error; and MAE: mean absolute error.

**Table 5:** R_pre/obs_ ratios for different PK variables of ofloxacin in different grades of RI populations.

Sr. No.	Study ID	Population	Administered Doses	C_max_ (μg/mL)			AUC_0–t_ (μg·h/mL)			CL (L/h)		
				PRE	OBS	R Ratio	PRE	OBS	R Ratio	PRE	OBS	R Ratio
			RI Population (PO Route)									
1-	Fillastre et al. (1987) [33]	Mild RI	200	1.91	1.52	1.25	22.32	28.79	0.77	8.16	5.88	1.38
2-	Fillastre et al. (1987) [33]	Moderate RI	200	1.59	1.55	1.02	25.50	34.33	0.74	7.01	4.9	1.43
3-	Fillastre et al. (1987) [33]	Severe RI	200	1.64	1.57	1.04	31.49	50.12	0.63	6.32	3.46	1.82

C_max_: maximal plasma concentration; IV: intravenous; AUC_0–t_: area under the plasma/serum concentration vs. time curve from 0–t; PRE: predicted; CL: clearance; OBS: observed; RI: renal impairment; and PO: per-oral.

## Data Availability

All the data generated during the research are reported in the manuscript.

## Supplementary Materials

The following supporting information can be downloaded at: https://www.mdpi.com/article/10.3390/pharmaceutics17091224/s1, Supplementary Figure S1: Sensitivity analysis of fraction unbound, specific intestinal permeability; Supplementary Table S1: Output of PK parameters as a result of sensitivity analysis of fraction unbound, and specific intestinal permeability.
