# Supplementary material for: A Comprehensive Physiologically Based Pharmacokinetic Framework of Ofloxacin: Predicting Disposition in Renal Impairment
Source: Pharmaceutics. 2025 Sep 20;17(9):1224. doi: 10.3390/pharmaceutics17091224 (PMC12473801; doi:10.3390/pharmaceutics17091224)
Supplement: Supplementary file 1 [file pharmaceutics-17-01224-s001.zip › pharmaceutics-3809078-supplementary.pdf]

## Supplementary Information

# A comprehensive physiologically based pharmacokinetic framework of ofloxacin: predicting disposition in renal impairment

Ammara Zamir [1], Muhammad Fawad Rasool [1*], Iltaf Hussain [2], Sary Alsanea [3], Samiah A. Alhabardi [4] and Faleh Alqahtani [5*],

[1] Department of Pharmacy Practice, Faculty of Pharmacy, Bahauddin Zakariya University, 60800, Multan, Pakistan; ammarazamir20@gmail.com (A.Z); fawadrasool@bzu.edu.pk (M.F.R)

[2] Center for Drug Safety and Policy, Xi'an jiaotong university, Xi'an PR China; Iltafhussain@stu.xjtu.edu.cn (I.H)

[3] Department of Pharmacology and Toxicology, College of Pharmacy, King Saud University, Riyadh, Saudi Arabia; Salsanea@ksu.edu.sa (S.A)

[4] Department of Pharmaceutics, College of Pharmacy, King Saud University, Riyadh, Saudi Arabia Salhabardi@ksu.edu.sa (S.A.A)

[5] Department of Pharmacology and Toxicology, College of Pharmacy, King Saud University, Riyadh 11451, Saudi Arabia; afaleh@ksu.edu.sa (F.A)

[*] Correspondence: fawadrasool@bzu.edu.pk (M.F.R), afaleh@ksu.edu.sa (F.A)

**Supplementary Figure S1: Sensitivity analysis of fraction unbound, specific intestinal permeability**

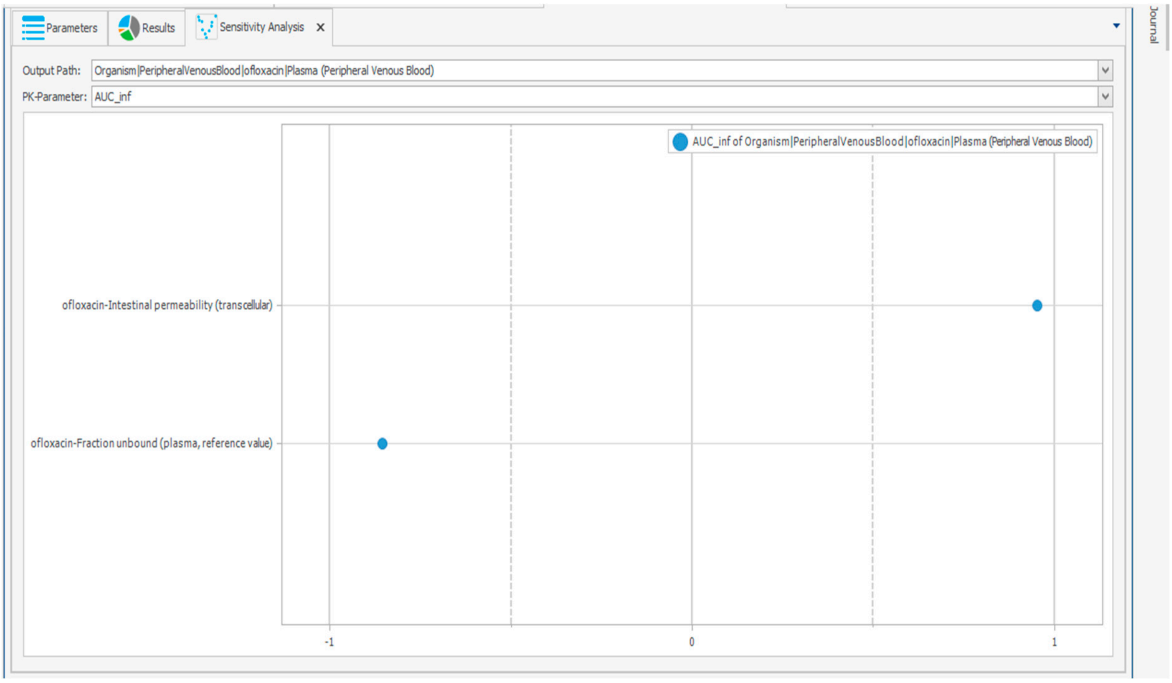


**Supplementary Table S1: Output of PK parameters as a result of sensitivity analysis of fraction unbound, and specific intestinal permeability**

| PK-Parameter | Fraction unbound | Specific intestinal permeability |
|---|---|---|
| **C_max** | -0.85 | 1.76 |
| **t_max** | -0.18 | -0.53 |
| **C_tEnd** | -0.85 | 0.37 |
| **AUC_tEnd** | -0.86 | 1.25 |
| **AUC_inf** | -0.85 | 0.95 |
| **MRT** | 0.01 | -0.64 |
| **Half-Life** | 0.03 | -0.47 |
| **% AUC (tlast-∞)** | 0.03 | -1.05 |
| **Total body clearance/F** | 0.85 | -0.95 |
| **Vss (plasma)/F** | 0.87 | -1.60 |
| **Vd (plasma)/F** | 0.88 | -1.43 |

$C\_{max}$: Maximal plasma concentration; $t\_{max}$: time to reach maximal plasma concentration; C_tEnd: observed drug concentration in plasma at the last recorded sampling time; AUC_tEnd: area under the plasma concentration–time curve from time zero up to the last measurable concentration; AUC_inf: total drug exposure over time, from time zero to infinity; MRT: Mean residence time; % AUC (tlast-∞): percent of extrapolated portion of the AUC beyond the last measurable concentration; F: Bioavailability; Vss: Volume of distribution at steady state; Vd: Volume of distribution

**Details of drug and population-related input parameters integrated in the PBPK model of ofloxacin**

**Drug-related parameters:** All physicochemical characteristics such as lipophilicity, fraction unbound, pKa, molecular weight, and solubility were added. Absorption models are built into the PK-Sim software, and only the specific intestinal permeability parameter was calibrated (in case of PO model calibration). Rodger and Rowland, and the PK-Sim standard method were used in the distribution section for the estimation of the partition coefficient and cellular permeability. Moreover, the elimination processes of ofloxacin, such as clearance by means of glomerular filtration and tubular secretion, were integrated into the renal clearance section. In the case of an oral model, an additional building block of formulation was used in which dissolved option was entered.

**Population-related input parameters**

The age, weight, height, and ethnicity of individual studies were taken from the respective studies, and the populations were built separately. In case of renal impairment, we have utilized the in-built population by embedding respective eGFR values and all the pathophysiological changes were not integrated separately. The range of values of eGFR were utilized in creating the population as mentioned in the respective study.
